# The natural history of conducting and reporting clinical trials: interviews with trialists

**DOI:** 10.1186/s13063-014-0536-6

**Published:** 2015-01-26

**Authors:** Rebecca MD Smyth, Ann Jacoby, Douglas G Altman, Carrol Gamble, Paula R Williamson

**Affiliations:** School of Nursing, Midwifery and Social Work, University of Manchester, Manchester, M13 9PL UK; Division of Public Health, University of Liverpool, Liverpool, L69 3GB UK; Centre for Statistics in Medicine, University of Oxford, Oxford, OX2 6UD UK; Centre for Medical Statistics and Health Evaluation, University of Liverpool, Liverpool, L69 3GS UK

**Keywords:** Qualitative, Interviews, Trialists, Research reporting, Recruitment, Trial protocols, Equipoise

## Abstract

**Background:**

To investigate the nature of the research process as a whole, factors that might influence the way in which research is carried out, and how researchers ultimately report their findings.

**Methods:**

Semi-structured qualitative telephone interviews with authors of trials, identified from two sources: trials published since 2002 included in Cochrane systematic reviews selected for the ORBIT project; and trial reports randomly sampled from 14,758 indexed on PubMed over the 12-month period from August 2007 to July 2008.

**Results:**

A total of 268 trials were identified for inclusion, 183 published since 2002 and included in the Cochrane systematic reviews selected for the ORBIT project and 85 randomly selected published trials indexed on PubMed. The response rate from researchers in the former group was 21% (38/183) and in the latter group was 25% (21/85). Overall, 59 trialists were interviewed from the two different sources. A number of major but related themes emerged regarding the conduct and reporting of trials: establishment of the research question; identification of outcome variables; use of and adherence to the study protocol; conduct of the research; reporting and publishing of findings. Our results reveal that, although a substantial proportion of trialists identify outcome variables based on their clinical experience and knowing experts in the field, there can be insufficient reference to previous research in the planning of a new trial. We have revealed problems with trial recruitment: not reaching the target sample size, over-estimation of recruitment potential and recruiting clinicians not being in equipoise. We found a wide variation in the completeness of protocols, in terms of detailing study rationale, outlining the proposed methods, trial organisation and ethical considerations.

**Conclusion:**

Our results confirm that the conduct and reporting of some trials can be inadequate. Interviews with researchers identified aspects of clinical research that can be especially challenging: establishing appropriate and relevant outcome variables to measure, use of and adherence to the study protocol, recruiting of study participants and reporting and publishing the study findings. Our trialists considered the prestige and impact factors of academic journals to be the most important criteria for selecting those to which they would submit manuscripts.

**Electronic supplementary material:**

The online version of this article (doi:10.1186/s13063-014-0536-6) contains supplementary material, which is available to authorized users.

## Background

That clinical research is important for the continued development of healthcare provision and the wellbeing of society is undisputed. Randomised controlled trials (RCTs) are considered the highest level of evidence on which to base healthcare decisions about healthcare interventions, making it essential that trials are performed and reported to the highest standards. Researchers have a responsibility to conduct the best research they can and publish accurate and unbiased results from it [1,2]. However, there is a large and growing body of evidence, across many specialties of healthcare, that the current conduct and reporting of RCTs is inadequate [3-6].

Problems associated with the conduct of research have been linked to the lack of a structured, practical and businesslike approach [7,8]. Problems include: trials failing to recruit the pre-specified number of participants [8], trials taking longer than expected to be completed [9], and problems associated with the collection of outcome data [10].

Another element of trial conduct that can be problematic relates to dissemination and reporting of findings. Underreporting or selective reporting of findings has been described as scientific misconduct [11]. Empirical research consistently suggests that findings from published research are more likely to be statistically significant than those from research that remains unpublished [12]. Not reporting whole studies based on the strength and direction of the trial results has been termed ‘publication bias’ [13]. Reporting bias can also occur within an individual study. For example, it may be that several outcomes are measured but only a selected subset of them are reported [14].

Published research articles generally provide an overview of the research questions, the methods used to determine their answers and the study results. Such articles rarely reveal the practical challenges encountered by researchers as the research progressed and how these challenges were managed. Thus, what is missing from the currently available literature is any account of the reality of undertaking research with all its challenges. Yet, it is important to know more about how trialists understand and carry out research and to explore how this might affect both research progress and reporting of findings. Moreover, there is a growing recognition that building a robust evidence base relies on trials being conducted to the highest standards, and it is widely accepted that those high standards cannot be assumed but have to be demonstrated by full and honest reporting of trials [15].

Qualitative research methods are ideal for studies that aim to explore previously unresearched topics and to identify perceptions and uncover meanings. At the time of designing this study, we scrutinised relevant databases and contacted key researchers but were unable to find any qualitative research that assessed the research process from the viewpoint or situation of the researcher. We therefore decided to investigate qualitatively the nature of the research process as a whole and factors that might influence the way in which research is carried out and researchers ultimately report their findings.

The study we report here was part of a larger study investigating outcome reporting bias in clinical trials, the ORBIT project [16]. Our focus here was on trialists’ real life experiences of carrying out and publishing findings from clinical trials across a range of clinical areas. The broad aim of ORBIT was to estimate quantitatively the prevalence and impact of outcome reporting bias in clinical trials. A nine point classification system for missing outcome data in RCTs was developed and applied to the trials assessed in a large, unselected cohort of Cochrane systematic reviews. Trialists were contacted and the reason sought for the non-reporting of data. A sensitivity analysis was undertaken to assess the impact of outcome reporting bias on reviews that included a single meta-analysis of the review primary outcome. Outcome reporting bias was suspected in at least one RCT in more than a third of the Cochrane systematic reviews examined [16].

The qualitative sub-study which is the focus of this paper was performed in two distinct parts. First, we compared original trial protocols with their linked subsequent publication(s) to identify the frequency of and reasons for selective outcome reporting and, for each trial where selective outcome reporting was identified, an *aide memoire* was produced, detailing both the pre-specified outcomes and the published outcomes as the focus for further discussion with the trialists concerned during semi-structured telephone interviews. The findings from this comparison have been reported elsewhere [17]. Second, trialists’ experiences of carrying out and reporting of research more generally were also explored through the interviews; it is this latter element of the sub-study which is reported here.

## Methods

We interviewed chief or lead investigators or co-authors of trials identified from two sources: trials published since 2002 covered in Cochrane systematic reviews selected for the ORBIT project [16]; and trial reports randomly sampled from 14,758 indexed on PubMed over the 12-month period from August 2007 to July 2008. Each interview was tape-recorded with the trialist’s permission, transcribed and anonymised. All interviews were conducted in English by one of the investigators (RMDS). Study methods used are described in more detail elsewhere [17]. The project was not required to be reviewed under the terms of the Governance Arrangements for Research Ethics Committees in the UK as it was considered the project came under the remit of audit/service development.

### Interview schedule

The focus of the interview schedule was on factors influencing the way research is carried out and how researchers report their findings. The schedule was organised sequentially, following the ‘natural history’ of a trial, and therefore had a pre-defined structure. The interview opened with questions about the main purpose of the trial in question, how the trial outcome variables were identified, and the use of and degree of the general adherence to the trial protocol. This was followed by questions relating to how the trial progressed and any associated challenges. Trialists were also asked to describe the process of writing up the findings and trying to get them published (including when papers were initially rejected). Additional information obtained at the time of the interview provided a descriptive summary of the characteristics of the trialist sample; for example, their previous research experience, the number of research sites involved in the trial under scrutiny, source of funding, level of statistical input to the trial design and analysis, and the planned and actual sample size. The interview schedule is as follows:

1. General discussion about the trial as a whole - time taken to complete the trial, role of team members, funding, objective/motivation for doing the trial, clinical context at the time relating to intervention
2. Regarding pre-specified outcomes - choice of pre-specified outcomes, pre-specify 1°/2°
3. Questions relating to reporting of outcomes (reported elsewhere)
4. Key questions relating to missing/partial reporting of outcomes (reported elsewhere)
5. Questions relating to the writing/getting paper published - roles of authors, statistician involvement, decisions made regarding publishing, decisions regarding choice of journal, journal decision, peer review
6. Role of the protocol - writing of the protocol, journal asked to see trial protocol, protocol used during trial/writing up period
7. Questions relating to publication bias more generally - ever not published (data/trial)
8. Reflecting on your experience of the trial - do differently, lessons learnt, regrets
9. Questions relating to experience - position at time, qualifications, trial experience
10. End.

### Analysis

Interviews were taped and transcribed, and qualitative analysis of transcripts was conducted [18]. The total number of interviews undertaken was governed by pragmatic reasons (that is, the number of trialists approached and agreeing to be interviewed), rather than theoretical considerations (that is, attainment of data saturation). The interview transcripts were compared and contrasted in order to elicit information regarding the individual trialists’ experiences, attitudes, perspectives, and opinions about the broad areas of trial design, conduct and reporting highlighted in the previous section. The analysis was led by RMDS but, to reduce possible interpreter bias and assess the plausibility and trustworthiness of the interpretation of the data, interviews were independently analysed early on by another member of the study team (AJ).The duplicate assessments were compared by going back to the original statements in the transcripts made by the trialists to validate both sets of interpretation. Two trialists requested, and were provided with, their interview transcript in order to add or clarify issues, and to ensure validity of the account produced. Each interview was given a unique identifier.

## Results

### Response rate

**Figure 1:**
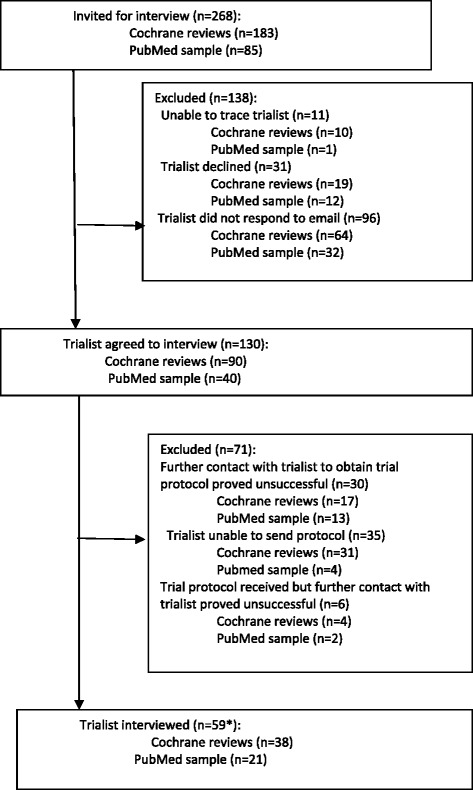
**Trialists eligible for interview.** *Includes chief or lead investigators or co-authors of trials identified from two sources: trials published since 2002 covered in Cochrane systematic reviews and trial reports randomly sampled from 14,758 indexed on PubMed over the 12-month period from August 2007 to July 2008.

A total of 268 trials were identified for possible inclusion in the qualitative sub-study: 183 published since 2002 and covered in the Cochrane systematic reviews selected for the ORBIT project and 85 randomly selected published trials indexed on PubMed. The response rate from researchers in the former group was 21% (38/183) and in the latter group was 25% (21/85). Overall, 59 trialists were interviewed from the two different sources (Figure 1) [17].

### Characteristics of trialists interviewed

Most interviews (48/59, 81%) were conducted with the trial chief investigator, eight of whom were also PhD students. A further eight interviews were conducted with the lead author and three with a co-author. Trialists were invited from 7 high-income countries (Australia, Canada, Germany, the Netherlands, New Zealand, UK and USA). All trials were performed after 1993. Trialists varied in their level of research experience; 32 (54%) had extensive experience and had been recruiting to trials for many years and had run their own trials. In contrast, six (10%) had no research experience. Just over a quarter (28%) said that no one with statistical expertise had been involved in the trial. Few trials (8%) recruited over 1,000 participants, most recruiting fewer than 100 participants (47%). Funding was provided by non-commercial sources for the majority of trials (56%).

Forty-three trialists provided the full study protocol for comparison with subsequent publication(s) by the ORBIT sub-study team. Despite the fact that many trialists highlighted the need for a formal, agreed, comprehensive protocol, there was wide variation in the quality of the protocols in terms of detailing the study rationale, proposed methods, organisation, and ethical considerations, and all lacked some key information such as a clear definition of the primary outcome or a description of the statistical methods planned for the analysis. There was no complete protocol available for the remaining 16 trials: six provided the ethics committee application, two the funding application, one a summary taken from the full protocol, and three extracts from relevant chapters of their doctoral theses. For one trial the protocol sent was simply a letter outlining a description of the trial sent to the funders. For two trials the full protocol was available, but not in English, and so an English summary was provided. For the remaining trial we obtained an abridged version of the protocol from the clinical trials.gov website [17].

Characteristics of the publication(s) were compared between trialists agreeing (n = 59) and those not agreeing (n = 209) to be interviewed. A higher proportion of trialists who did not agree to be interviewed were funded by industry (82% vs 18%). There was no evidence of association between a trialist agreeing to be interviewed and the sample size of the trial.

The median publication year was 2005 (range 2002 to 2008). In all but one case interviews were performed with one trial investigator from each trial; for one trial we interviewed the chief investigator, the lead author and statistician simultaneously at their request. Interviews lasted on average 56 minutes (range 19 to 96 minutes).

### Major responses around the natural history

A number of major but related responses were identified regarding the conduct and reporting of trials. In presenting these we have ordered the data to reflect the natural history of conducting a clinical trial, namely:

- Framing the research question
- Defining key outcomes
- Role of the study protocol
- Problems with conduct of the research
- Issues around getting the research published.

### Framing of the research question

All trials were conducted as a result of identification of a clinical need or where clinicians were in clinical equipoise. Some of the trialists were experienced researchers and had themselves performed previous empirical research, as a consequence their research questions were “in part generated through our earlier work”.

Others, however, identified voids in the published research literature where little or no actual research had been performed. As a consequence, this gave the trialists the basis on which to formulate their research questions:

“There were virtually no control studies of the intervention, so it seemed to be a wide open area where there is a great need to pursue intervention” (31).

“So I discussed it with colleagues that you know I wonder if this isn’t something that should be tested. We looked at the research and we couldn’t find anything published that actually did a test” (6).

One trialist used the term equipoise spontaneously, talking about it at an individual level and the wider community level: “We had equipoise on this subject, and we felt that other people did not have equipoise. So there was an international move to change policy, and introduce an intervention that we felt had not been tested and evaluated adequately” (27).

### Defining key outcomes

The responses from the trialists clearly showed that pre-specified outcome variables were generally identified from either clinical practice or expert opinion:

“It is a very clinical question and they are experienced clinicians who know what’s important in the clinic” (7).

“Well we looked at the literature and we also spoke directly to [name] - he has done more trials with the population than anyone else, and so he and our colleagues met with him and got his advice about what should be measured” (37).

We asked trialists if there was a formal consensus process in their clinical field for identifying outcomes. However, very few (n = 2) were able to base decisions about what constituted key outcomes, as for most they often did not exist. Others identified the role systematic reviews play:

“I think one of the challenges with the protocol development is us defining what an appropriate outcome was, and I think at the time when this was written, I think that was about 2003, there was not yet a consensus based criteria. So at the time that was part of the challenge in figuring out what was a reasonable outcome that was feasible for the study that we were planning to conduct but actually had some validity” (56).

“According to Cochrane analysis we must take into account not only [outcome] and problems of patients, but also quality of life and therefore we added quality of life in our measurements” (2).

There was, though, evidence of some degree of guidance or informal consensus within individual specialties which was provided by two trialists:

“Yes there are, there are recommendations or guidelines. And there is some latitude within those guidelines” (15).

“So in the absence of a consensus statement [....] there probably is informal consensus” (53).

In addition to lack of consensus about whether they were informative, relevant or important, one trialist recognised different factors were dominating the selection of outcome variables, for example ensuring the trial would be funded:

“I think even when we were designing the protocol, there was less consensus over whether that was an important outcome. I think partly because if you had omitted that I think some reviewers might have said: ‘wow you are not measuring [outcome]’. That being said there is a vast amount of literature showing that it’s of completely no relevance, but it was a practical decision to make sure we got money. So I think we were pretty ambivalent about its inclusion, and certainly once we had analysed it we thought you know, it didn’t really add much to the information coming out from the paper, so we didn’t include it” (56).

### Role of the study protocol

The role of the protocol was discussed in the interviews. Authors reported differences in application of, and adherence to, the protocol during the conduct of their trial. Some, unsurprisingly, used the protocol to ensure adherence to study procedures and to assist in writing the final publication. Conversely, in some cases it was apparent the protocol was not referred to once the trial was in progress:

“Yes, the physicians that are participating in the trial, yes they, they do have the protocol and they are expected to follow the protocol clearly” (41);

“You write the protocol you spend months doing it, and you have got a really good grasp of it and then as the trial progresses the protocol fades, and it’s not just in this trial, but in other trials” (59).

Regarding journals requirement of submission of the trial protocol alongside the manuscript, three trialists had been asked to provide their trial protocol as part of the process. All journals had an impact factor of at least 30.

### Problems with conduct of the research

Where the trialists identified problems with trial conduct, these were mainly surrounding recruitment difficulties and the target sample size not being met. Thirteen trialists reported that recruitment was more difficult than expected, resulting in fewer participants being recruited, of which four acknowledged this in their reporting, reasons for which are unclear. Recruitment difficulties were variously explained by potential participants’ lack of motivation to join the trial and over-estimation of recruitment potential:

“We actually got a very high take-up rate, but treatment as usual made the trial a bit unpopular with some people, I think we had 60 odd who refused. They didn’t want to be in a trial where there was a 1 in 3 chance of getting treatment as usual” (20).

“We had some difficulty with enrolment, I think because there were less patients available than we had envisaged at the beginning” (35).

The interviews also highlighted instances where recruitment problems led to early trial closure or prompted changes in the research design:

“We were running out of money and the recruitment was still quite slow, so we looked at the collection of subjects and we found no difference, it just didn’t seem worth going on. Also, the analysis was driven by an opportunity to present the data at a conference and so we analysed what we had at that point, it didn’t seem worth continuing” (5).

“We changed the follow up interval [from 24 months to 20 months] because it took us longer to do recruitment than we thought it would. Also we wanted to finish the study in the time line we had funding for and decided that probably those four months weren’t going to make a whole heck of difference, so we compressed the timeline” (4).

Trialists also highlighted problems among trial recruiters themselves. Although equipoise was generally not expressed overtly, it appeared that clinicians were always not uncertain about the treatment effects; and it was apparent that some were unable to recruit participants effectively in situations where they had some doubts:

“It was more difficult to recruit than we had imagined. We realised that the nurses were recruiting in a very sort of naive way – in that they were selecting women for the trial who they thought the trial would work for. So they had a preconceived idea in their head although we had spoken to them and provided them with information” (37).

Interviews also highlighted that trial recruitment was being stopped early based on the statistical significance of the results at the interim analysis:

“We finished earlier because we found that there were statistically significant differences and therefore we didn’t continue with more than 28 patients. If there were no statistical differences maybe we would have asked our agency to continue funding, but we found differences and it was good for us, very good news for us” (2).

“The initial power calculation was for 60 patients and after 30 patients when we analysed the data we did see, you know a trend, benefit at 6 months so we felt let’s publish and go for it. If I didn’t see any differences I would have probably continued to enrol patients – if I didn’t see anything to publish” (34).

For six trials, although the sample size was achieved it was not without recruitment difficulties, mostly linked to poor planning of the trial. Trialists reported problems with participant eligibility, either because of inclusion criteria being too tight (n = 3) or because changes in clinical practice led to changes in the eligibility of certain populations (n = 1):

“The percentage of people who actually agreed was remarkably high, I think two thirds of everybody approached agreed to be in the study. The problem was finding them to approach them and that required a fairly intensive effort. We had to have teams of people waiting in the medical offices for a person of the right age, and that turned out to be much harder than we had thought, and we eventually had to add more staff and add more clinics to reach our sample size” (15).

“When we did the pilot study we were recruiting from the emergency department and were able to get phone consent from parents when they weren’t present in the emergency department. Between that trial and this trial the hospital changed its rules and we had to have in-person signed parental consent before we could start the assessment and intervention with the children, and that made things a lot more difficult in terms of recruitment and enrolment” (13).

In addition, trialists reported practical difficulties in running a trial, either because of difficulties collecting outcome data (n = 4), problems associated with the randomisation process (n = 1), difficulty recruiting research staff (n = 1) and revisions required post-pilot phase (n = 1). Concerns were also raised regarding the level of adverse outcomes, even when lower than expected:

“Even though we had said a 20% mortality for study suspension or closure and we were not at that, it was still felt that probably the number was too high and there was just general concern about the extent of early mortality in this study” (58).

“Just looking at the raw data without knowing what treatment they were assigned, you could see there was poor efficacy overall. So at that point we got someone independent (statistician) to look at the data and there was absolutely no difference at all. Then what we did was say if there was a tiny difference do a power calculation on how many patients you need to recruit to see a small difference and you need something like 500 – so we knew there was no point recruiting further patients. Together we, with the PI, we decided there was no point going on with this. We discussed it with the statistician and we all agreed there was no point continuing because there was such a strong signal that it was negative” (46).

This trialist indicates that although they were blinded to intervention allocation they nonetheless had access to outcome data. The trialist goes on to describe the approach taken by the study team, which was to decide as a group to stop the trial.

In about half the trials (n = 30) there were no issues with recruitment or retention, in three cases the sample size being achieved earlier than expected. Some groups performed pilot studies, which assisted in predicting and preventing problems prior to the trial starting. One group had forecast recruitment difficulties but decided to go ahead anyway, in order to secure funding (n = 1):

“The grant they gave us was the maximum so we put together a proposal that could make best of that knowing it was unlikely that we would achieve what we wanted to achieve within that money, and I think the funder knew that as well, so they were very forthcoming when we asked for an extension” (30).

### Issues around getting the research published

Trialists were asked about the process of publishing the primary paper from the trial. Thirty-five (59%) trialists had their manuscript accepted at first submission; twenty-three had their manuscripts rejected at first submission, one trialist could not recall if accepted at first submission. The most common reasons given by the trialists for rejection at first submission were related to study design (n = 13), non-significant results (n = 3), not fitting with the topic areas of the journals approached (n = 2), or trialists unable to recall reason/no reason by journal given (n = 5).

Trialists reported that the impact factors of journals and their kudos within the clinical specialty where they worked generally dictated which journal they approached first for publication: “It’s the most prestigious journal in our field; it has the highest citation value of any journal in our field” (53). Some authors were forced to opt for lower impact factor journals due to methodological issues (that is, ending recruitment early, or failing to recruit the pre-specified number of patients): “For the kind of journals which are high impact factor; unfortunately they wouldn’t take a paper like this for two reasons – one which is valid is that it was stopped prematurely and because it’s not positive and negative trials are quite difficult to publish. So the high impact journals we thought probably wouldn’t take it” (46).

Several trialists reported feeling restricted by word limits imposed by journals and highlighted the implications for reporting of all outcomes:

“The reviewers do not want us to report on all the outcomes, if the journals would allow us to publish longer papers and were interested in reading longer papers I would be happy to report on all possible outcomes. Secondly, there is limitation in terms of pages, in terms of tables, in terms of words that you can submit” (41).

For one trialist there was little guidance given by the journal regarding what aspects of the paper were to be omitted, and therefore the reporting of results was not affected:

“We were asked to cut the paper, they did not give us specific direction on what to cut they just told us to cut it, none of the analysis were removed” (13).

A number of trialists cited problems with journal peer review as influencing the final version of the manuscript, one in particular citing the problem of competing interests amongst peer reviewers:

“My recollection was we had three very positive reports and one that was very bad, and I know who that was from, that was one of our competitors, but it was so obvious who it was from” (38).

Other trialists acknowledged issues around study design and inadequate sample size as problematic for publication:

“I don’t think the referees liked the fact we didn’t have a placebo, so that was a design aspect. And the design was actually forced on us by the funders. Because this was a pragmatic trial, we actually put in for a placebo controlled efficacy trial, and the [funders] told us we should be doing an effectiveness or cost effectiveness trial” (20).

“I think because the sample size was relatively small, and it was a negative study so they were concerned it wasn’t adequately powered to really answer the question” (5).

In addition, some trialists thought that the lack of statistical significance of their findings impacted on the decision by the journal not to publish:

“They declined it because it was a negative finding” (36).

“We felt, if you’ve got a negative trial that perhaps people were less interested” (59).

One trialist discussed several issues regarding rejection of their work, including that because the results were negative the journal editor vetoed publication, so giving publication bias as a suggestion of what had occurred:

“It was actually quite disappointing, we went through two rounds of reviews and after an extensive re-write they rejected it, and I think the line was – you know that your work is interesting but fundamentally your results are negative. It’s like I know that, so it certainly was an exercise in learning more about publication bias. I was surprised at how blatantly the editor stated it, I mean he just said it’s interesting but your results are negative. At the time I was more irritated because he had wasted our time, because you know he would have known that reading the abstract in my first submission then we could have just ended this, it just wasted three months of my time” (56).

In contrast, in considering their experience of getting their work published, a number of trialists described the constructive impact peer review had had on their manuscripts and felt that feedback ensured a more balanced manuscript:

“Journal reviewers are quite helpful in getting a better finished product. Reviewers ask important questions and help to clarify things that as an author you have thought were perfectly clear” (5).

“I think we probably, because the effect size was larger in the [intervention] group, we probably made it sound a little bit more positive in terms of our findings, that they wanted us to make sure that we toned it down to say that we really didn’t find many differences and that both of these treatments were pretty comparable in a lot of ways. I think that’s often the case, reviewers want to make sure you don’t claim more from your data than you can actually support” (23).

### Previous experience around publishing trial findings

All but two trialists (due to time constraints of interview, n = 1; not performed a trial previously, n = 1) were asked directly whether they had ever chosen to not publish previously conducted trials. Over half the trialists (n = 33) stated that they themselves had never chosen not to publish data, even when the findings were unexpected, negative or unfavourable:

“We have had a couple of failed studies, there was one study, it was supposed to be a huge trial, looking to recruit 1,800 men, and we closed after 2 years with 35 randomised, so clearly you are not going to get anything useful out of that, but you know I badgered and badgered and eventually we managed to write a research letter to an international journal, where we just presented the baseline characteristics of the patients that joined and we talked about the randomisation of patients and we presented some anecdotal information about why we thought the trial failed” (7).

Three of the 33 trialists had, however, been involved as site investigators in industry-funded trials that were never published, one trialist reflecting on their experience as follows:

“I can think of at least one trial that has not been published. I was not the PI, I was just a site investigator, that was an industry sponsored trial that has not been published. Not only was it a negative result but the study was stopped early because of safety. And that has not been published” (26).

Those authors who had failed to publish any findings from a trial (n = 16) cited many reasons: negative study findings, lack of time, lack of resources, recruitment problem, rejection by journals, unclear results, or failure to complete the trial. Five trialists had published the primary, but not the secondary, outcome results:

“I have certainly not published as many papers as we could have, but not deliberately – it’s usually lack of the soft money that by the time you are done the grant has run out and then you have got a new thing starting and you know one person can’t publish all the papers” (4).

“The major problem was we were never able to recruit enough subjects for the study, and so the study got terminated and we were never able to make use of the data” (40).

Three trialists were in the process of writing up their findings at the time of the interview, one of whom highlighted the potential for apathy in writing up negative findings and hence a potential significant time lag between completion and publication of negative trials:

“Well I am actually struggling with it right now. I have an implementation trial and the results are negative and I am pushing myself along, I am taking longer to put it in than I should, and the paper is now drafted and we are working on the final references so it is going to go in, but you know I have to push myself pretty hard – I feel bored. And then I think sometimes people are really wedded to a hypothesis and they just don’t want the negative results out there” (15).

## Discussion

Our study adds to the scant literature on trialists’ experiences of performing and publishing clinical trials in healthcare. We set out to explore researchers’ experiences and to identify which factors appeared to shape their experiences. We have provided an insight into the nature of the research process from the researcher’s perspective and our results confirm that the conduct and reporting of some trials can be inadequate. Nonetheless, our work has identified that aspects of clinical research can be inherently challenging.

Our work has demonstrated that a substantial proportion of trialists identify outcome variables based on their clinical experience and knowing experts in the field. Thus our results concur with a previous study showing insufficient reference to previous research in the planning of a new trial [19]. There appeared to be a general lack of consensus in most clinical settings regarding choice of appropriate outcomes. Problems associated with lack of consensus relate to lack of clarity regarding choice of outcome measures [20,21], outcome reporting bias [14] and selection of outcomes that may not be meaningful to clinicians [22] or health service users [23,24]. Using standardised sets of outcomes to improve the standard of reporting and make it easier for the results of trials to be compared, contrasted and combined as appropriate is the focus of the COMET (Core Outcome Measures in Effectiveness Trials) Initiative [25,26].

Recruitment of participants into trials is critical for successful trial conduct. Not reaching the target sample size can mean that results are less reliable and so less useful in clinical practice. However, extending the timeframe for recruitment generally increases the costs of trials (the longer-term impact being that fewer trials can be conducted). However, recruiting participants to trials can be challenging [7,27,28]. Our findings are consistent with previous studies revealing problems with trial recruitment, potential participants’ preferences, over estimation of recruitment potential and recruiting clinicians not being in equipoise. Work by others [29] has revealed clinician barriers relating to time constraints (for example, time pressures from usual clinical practice as well as staffing and staff training), lack of research experience in clinicians, adverse effect on doctor-patient relationship, perceived conflict in their role as clinicians and researchers and perceived burden of trial for patients including travel distance and costs have all adversely affected recruitment to clinical trials.

Work has been carried out to evaluate the relative effectiveness of recruitment strategies for participation in RCTs [27]. Thirty-seven studies that compared methods of recruiting individual study participants into an actual trial or mock RCT were included. Strategies to increase participant awareness of the health problem under study, attendance at educational sessions, the addition of health questionnaires, or a video about the health condition, and monetary incentives were all found to improve recruitment. These findings lend further support to feasibility assessments and pilot trials being a potential solution to this problem.

Conversely, the motivation of clinicians to participate in clinical trials in the UK has also been qualitatively studied [30]. This work reports clinicians consider having an interest in the research question, intellectual curiosity and potential benefits to patients (including access to treatments or drugs, and closer monitoring) as opposed to payment for involvement are the most important factors [30].

High-quality protocols facilitate proper conduct, reporting, and external review of clinical trials [31]^.^ However, our results confirm that the quality of trial protocols can be substandard. We found wide variation in the completeness of protocols, especially in relation to the study rationale, the proposed methods and trial organisation, and ethical considerations. There was also variation in the precise definition and scope of trial protocols; 16 of our trialists could not provide a completed comprehensive document that included important information relating to study design. It is now widely acknowledged that every clinical trial requires a complete and transparent protocol to facilitate sound trial conduct [32]. However, previous work has confirmed the widespread deficiency in protocol content [33-35]. In response to these shortcomings the SPIRIT (Standard Protocol Items: Recommendations for Interventional Trials) international initiative was developed [31,36]. This initiative aims to improve the quality of clinical trial protocols by providing a minimum set of scientific, ethical, and administrative elements that should be addressed in a clinical trial protocol. The mechanism of journals requesting protocols to be submitted alongside the manuscript is one way of allowing a comparison of what was planned and what was actually done.

Our trialists considered the prestige and impact factors of academic journals to be the most important criteria for selecting those to which they would submit manuscripts. This confirms work by others where the constituency of the readership, whether the journal usually published articles on the topic, and the likelihood of manuscript acceptance were also considered key criteria [37].

Peer reviewed publication is the final, essential step in any research project, providing legitimisation and credit for the work that has been done [38]. We found there was a perception that journals were more or less interested in studies depending on the direction of the findings and the presence/absence of a so-called ‘negative’ result. In addition, several trialists experienced difficulties when writing up and getting their manuscripts published because of word limit restrictions for paper journals. Electronic journals allow authors to exceed the usual paper journal word limits. In addition, open access allows researchers to read and build on the findings of others without restriction. The NIHR HTA programme has a high publication rate and is the world’s first health research funder to publish comprehensive accounts of all of its research. The launch of the NIHR Journals Library follows the success of the HTA journal and aims at ensuring other participating NIHR programmes publish their research. All results are reported, whether they are positive, negative or neutral, minimising publication bias and maximises the usefulness of data in subsequent studies [39]. Open access also means that teachers and their students have access to the latest research findings throughout the world, so acting as an important educational tool [40].

Further difficulties associated with the editorial process were connected to peer review. There was evidence from the trialists’ accounts that competing interests amongst peer reviewers was a potential issue. Conflicts of interest by peer reviewers need to be taken more seriously by all [41]. Reviewers need to declare all conflicts of interest, since competitive issues or personal relationships can lead to important and less obvious bias.

Some of our trialists recognised the two principal functions in medical journal publishing as: to select reports for publication that meet scientific quality standards; and to improve presentation of research during the process of revision [42]. Several investigators have studied changes to manuscripts during the editorial process and its impact on manuscript selection, these being mostly related to changes in readability [43], and the adequacy of statistical reporting [44]. Our trialists confirmed that the peer review process had ensured their final published manuscript was a balanced account of the work performed. For example, it has been reported that authors often overemphasise study findings in an attempt to impress the journal with their importance [3] and the trialists in this study acknowledged that the peer review process allowed for the recommendation that the findings were publishable if overstated conclusions were toned down.

A substantial number of trialists disclosed that there had been occasions when they had not published previous research findings. Reasons provided included negative study findings (sometimes related to not achieving the sample size), or lack of time and resources. Our qualitative work is consistent with previous quantitative findings [14] revealing that not reporting a study based on the strength and “direction” of its results - termed publication bias - is common. Trial results should be published whatever the outcome of the trial. However, it was apparent from trialists’ accounts that they were often genuinely unaware of the potential problems their decisions not to publish could cause. Importantly, we found no evidence of intentional wrongdoing by the trialists, but rather scientific naivety. Moreover, it appeared that the process through which trialists are required to journey is one of negotiating hurdles and responding to challenges that often cannot often be anticipated. Trialists sometimes have to adapt their research plans, in order to make their way through the complex research process, and may do so with little in-depth knowledge or formal training in research methodology.

### Strengths and limitations

The strength of our study lies with its use of qualitative interviews to provide real insights into the lived experience of doing research across its natural history, from the perspective of the investigator. Our study adds to the evidence base on how trials are designed and conducted in practice [45-47]. Our findings are in accord with the issue of trial management being inherently complex. To review the literature, develop a protocol, apply for funding and design case report forms requires lengthy consultations and a considered approach [7]. Studies such as ours exploring all phases of clinical research offer transparency about the process which, in turn, may help to identify aspects where procedures or education will help to reduce bias, improve credibility of results and cultivate the efficient management of trials.

We have presented the experiences of only those who agreed to be interviewed and who, perhaps, represent researchers with a particular interest in adhering to sound research procedures. As noted earlier, a significant proportion of those declining participation were funded by the pharmaceutical industry, which may itself represent a source of bias. Nonetheless, our trialist participants did come from a wide range of clinical specialties, research experience and countries, supporting the generalisability of our findings. In addition, we acknowledge the low acceptance rates from both sources of recruitment (21% from Cochrane reviews selected for the ORBIT project and 25% from randomly selected published trials indexed on PubMed). A number of trialists declined our offer of interview without giving a reason, which may mean that the true picture is worse than described; in addition there were those who were unable to locate the trial protocol - itself suggestive of poor research practice [17].

## Conclusion

Our work has identified that aspects of clinical research can be inherently challenging, in particular with regard to establishing appropriate and relevant outcomes to measure, the use of and adherence to study protocols, recruitment of study participants and the reporting and publishing of the study findings. To ensure clinical relevance and minimise questionable research practices, it is crucial that measures are taken to optimise the reliability of studies that are conducted. Potential solutions to the problems associated with poor trial design, conduct and substandard reporting practices include: the development of systems to support and train researchers in trial methodology and processes, such as those being provided by the UK Clinical Research Network [48], and the development of research ‘toolkits’ such as The Clinical Trial Toolkit [49] and The Guide to Efficient Trial Management [50]. Additional means to improve trial conduct includes support from the UKCRC Registered Clinical Trials Units (CTUs). Since 2007, all UK National Health Service trials are strongly encouraged by funders to involve CTU expertise. The CTUs have the capability for centrally coordinating multicentre clinical trials, as well as for trial design, data management, and analysis [51]. Improving the reporting quality of trials should include: registering clinical trials at their inception in a publicly accessible database [52,53]; the adoption of evidence-based reporting guidelines, such as the CONSORT statement [54] and the PRISMA statement [55]; an evidence-based minimum set of reporting items in systematic reviews and meta-analyses, in addition to research ethics committees having a clear remit to ensure the responsible conduct of research; and insistence by journal editors on the submission of a trial protocol alongside the manuscript [56]. Implementation of such strategies to support trial management will not only benefit trialists and the broader research community, but also the end-users of trials, namely the patients they seek to serve.

## Abbreviations

CTU: clinical trial unit
RCT: randomised controlled trial
